# A Simple Deterministic Model of Protection and Cost Benefits from Vaccinating Indian Cattle Against Infectious Bovine Rhinotracheitis

**DOI:** 10.3390/pathogens14090955

**Published:** 2025-09-22

**Authors:** Bhaskar Ganguly, Sarvesh Tayshete, Priyabrata Pattnaik, Nyayapati Sunil Narayana Bhargav, Anand Kumar Kanakasapapathy

**Affiliations:** Indian Immunologicals Limited, Rakshapuram, Hyderabad 500032, India

**Keywords:** Infectious Bovine Rhinotracheitis, Bovine Herpesvirus-1, vaccination, control, modeling, simulation

## Abstract

Infectious Bovine Rhinotracheitis (IBR) is endemic in India, causing significant losses to dairy enterprises. Until recently, the unavailability of an indigenously manufactured vaccine and the high cost of imported vaccines limited national vaccination efforts. However, an indigenously developed and manufactured inactivated DIVA vaccine has now become available. The exact strategies that other countries have employed for the successful control of IBR may not be applicable in India due to the differences in the production systems and the social values. Hence, we have employed linear deterministic modeling to study the benefits, both in terms of the protection of the animals from the disease and the costs, of vaccination against IBR towards proposing an optimal strategy for immunization-based control of the disease in India. Our findings emphasize the need for proper vaccination practices, appropriate farm biosecurity measures, and biannual re-vaccinations to achieve the desired endpoints in a vaccination program. Based on our findings, a vaccination program aiming for primary vaccination with two doses followed by continuing bi-annual re-vaccination with a single dose to achieve 70% vaccination coverage in the cattle population can be recommended for the control of IBR in India.

## 1. Introduction

India tops the world in cattle population and milk production. India also experiences the greatest losses from dairy diseases in the world [1]. Amongst these diseases, some commonly occurring reproductive disorders dampen fertility, prevent conception, lead to postpartum complications, increase inter-calving periods, reduce healthy calf crop, and lower overall lifetime productivity [2]. Interestingly, many of these reproductive disorders have been associated strongly with Infectious Bovine Rhinotracheitis (IBR), which is caused by Bovine Herpesvirus-1 (BoHV-1) and is endemic in the [3]. It was found that 83% abortion cases, 76% metritis cases, 83% repeat breeding cases, and 65% retention of placenta cases were seropositive for IBR [4]. Seropositivity for BoHV-1 has also been associated very strongly with significant losses in milk production and body weight gain [5,6]. Association does not necessarily infer causation; however, sufficient evidence is also available to show that high IBR prevalence contributes actively to these losses [7,8,9]. The average prevalence of IBR across India has been reported at more than 33%, with some areas recording positivity exceeding 60% [10]. Reliable estimates of per-animal losses due to IBR are not available from India. Previously, a study from Turkey estimated the average financial loss from BoHV-1 infection at US$379 per dairy cow [5].

The need for a nationwide vaccination program against IBR has been voiced on several occasions [10,11], but the unavailability of an indigenously manufactured vaccine or high prices of imported vaccines have proven limiting [12]. Only recently, an indigenously developed and manufactured, inactivated DIVA vaccine has become available [12,13], which may be used to control the disease. Most countries that have successfully controlled IBR have employed inactivated DIVA vaccination and test-and-cull strategies [14,15]. Understandably, the exact strategies may not be applicable in India due to the differences in the production systems (viz., purpose, animal-type and breeds, rearing practices, etc.) and the social reservations against the slaughter of cattle [16].

In the present study, we employ linear deterministic modeling to study the benefits, both in terms of the protection of the animals from the disease and the costs, of vaccination against Infectious Bovine Rhinotracheitis towards proposing an optimal strategy for immunization-based control of the disease in India.

## 2. Methods

Three different sets of parameters were modeled, and several assumptions were made for developing the models based on extant literature and experiences with other vaccination programs for livestock.

First, the vaccine coverage required to achieve herd immunity was calculated under different scenarios. Basic reproduction number (R_0_) of BoHV-1 was assumed at five different levels of 3, 4, 5, 6, and 7. Vaccine efficacy was assumed at three levels of 70%, 80%, and 90%. Herd immunity threshold (HIT) and vaccine coverage required to achieve herd immunity threshold were calculated as:

(1) $$HIT=1-\;\frac{1}{R_{0}}$$

(2) $$Required\;vaccine\;coverage=\frac{HIT}{Vaccine\;Efficacy}$$

Thereafter, the reduction in prevalence of the disease with vaccination was modeled assuming three different vaccine coverage levels of 60%, 70%, and 80%, respectively. A prevalence-reduction model was assumed where prevalence reduces proportionally to the immunity gap each year, i.e.,

*P_t_*_+1_ = *P_t_* × (1 − *r*) (3)

where

*P_t_* is the prevalence at year *t*, and

*r* is the reduction factor and is capped at 0.5 (50%)

Prevalence of the disease was assumed at four different levels of 18%, 35%, 45%, and 60%; HIT was assumed at 50%; and effective immunity was calculated as:

Effective Immunity = Coverage × Vaccine Efficacy(4)

If effective immunity met or exceeded HIT, a 50% reduction in prevalence was accommodated per year, and if effective immunity was less than HIT, a 10% reduction in prevalence per year was made. This is a simplified deterministic model, not accounting for stochastic effects, latency, or reactivation. Booster effects were modeled based on additive assumptions; each dose of booster increased vaccine efficacy by 5% with a capping at 90%.

Finally, cost–benefit effects were modeled under three different levels of vaccine coverage, i.e., 60, 70, and 80% with booster effects. For this part of the analysis, the population size was assumed to be 10,000, and the prevalence was assumed to be 33%. Since estimates of losses due to IBR in India are not available in absolute terms, a relative cost approach was used. Losses due to the disease were assumed at 20*x*, the cost of vaccination at 2*x*, and the cost of re-vaccination/booster at 1*x*, where *x* is an arbitrary unit of currency. Further, total cost and total benefit were calculated as:

Total Cost = (Cost per Dose + Booster Cost) × Number of Animals Vaccinated(5)

Total Benefit = (Total Cases without Vaccination − Total Cases with Vaccination) ×  Economic Loss per Case per Year; where(6)

Total Cases without Vaccination = Initial prevalence × population × years(7)

Total Cases with Vaccination = Sum of annual prevalence × population(8)

Net Benefit = Total Benefit − Total Cost(9)

The total yearly costs were added to obtain the cumulative investment. Similarly, yearly net benefits were added to obtain cumulative benefits. The ratio of cumulative benefits to cumulative investments was expressed as cumulative return on investment (ROI).

All models were simulated and visualized in Plotly (version 3.1.0) [17] (Supplementary File S1).

## 3. Results and Discussion

**Figure 1:**
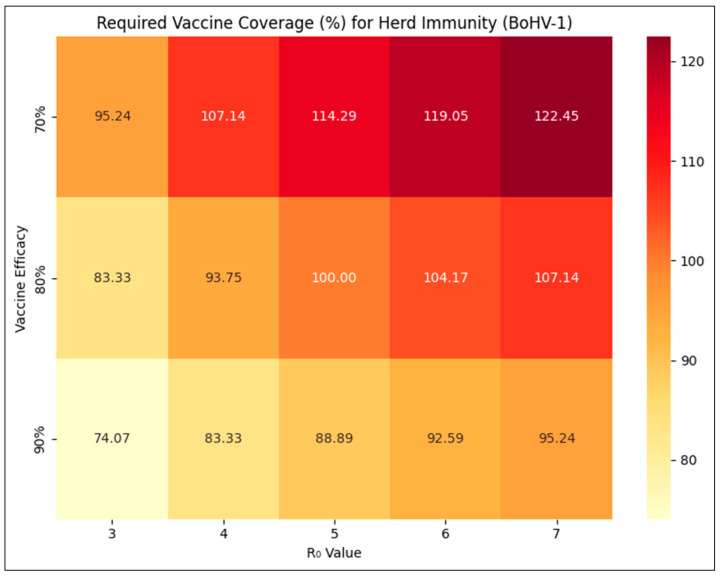
Vaccine coverage (%) required to achieve herd immunity threshold for BoHV-1 infection at varying levels of basic reproduction number (R_0_) and vaccine efficacy.

The percent vaccine coverage required to achieve the herd immunity threshold at varying levels of R_0_ and vaccine efficacy is shown in Figure 1. R_0_ was assumed at five different levels of 3, 4, 5, 6, and 7. R_0_ of BoHV-1 is not known in Indian conditions, but values of R_0_ ranging from 3.2 to 7 have been estimated for cattle in other parts of the world [18]. Given the guidelines for IBR vaccines [19], the efficacy of any licensed vaccine is unlikely to be less than 80%. The recent, indigenously developed DIVA vaccine against IBR from India [13] has also been shown to be 90% efficacious [12]. However, factors such as handling of the vaccine or individual animal responses may dampen immune response and efficacy [20]. Hence, vaccine efficacy was assumed at three different levels of 70%, 80%, and 90% for determining the required vaccine coverage.

As expected, the required vaccine coverage increases as R_0_ increases or vaccine efficacy decreases. Coverage levels exceeding 100% are practically absurd and cannot be achieved. Even in the dedicated and intensive national control program for FMD in India, achieving 80% vaccine coverage was initially considered aspirational and high [21]. For newly introduced vaccinations, coverage is likely to remain low at first, with values of about 60% being more realistic [22], and increase gradually as diffusion of innovation [23] takes place and policy gains momentum [24]. Hence, proper vaccination practices that ensure greater efficacy and appropriate farm biosecurity measures that reduce transmission and result in lower effective R_0_ remain essential, especially so for high-prevalence areas, to achieve herd immunity, albeit realistically lower vaccine coverage rates.

**Figure 2:**
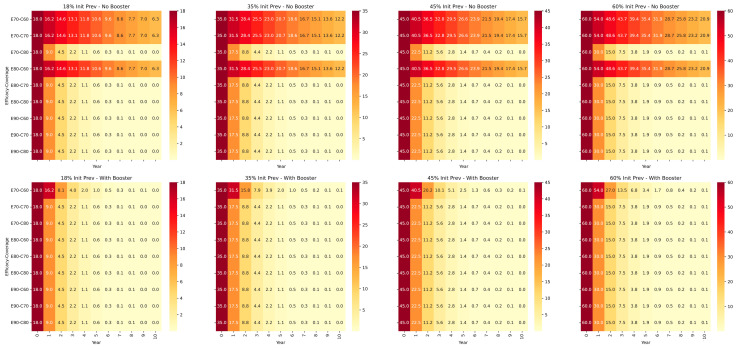
Decrease in seroprevalence rates of BoHV-1 over 10 years, assuming initial seroprevalence (Init Prev) rates of 18%, 35%, 45%, and 60%, respectively. Two different scenarios were simulated; the top half shows the decrease in seroprevalence when re-vaccination is annual and six-monthly boosters are not used. The lower half panel shows the decrease in seroprevalence with six-monthly boosters, assuming 5% increase in vaccine efficacy with each booster dose, capping at 90%. Three different levels of vaccine coverage of 60%, 70%, and 80% were assumed.

The seroprevalence of IBR varies a lot across different regions within India. Previously, the country has been divided into five zones based on IBR positivity of nil (0%), up to 17.95%, 17.95–34.32%, 34.32–46.67%, and 46.67–61.51% [10]. For the ease of calculation, these values have been rounded-off to 18%, 35%, 45%, and 60% initial seroprevalence levels, respectively, for the purpose of modeling the response of the population to vaccination with or without 6-monthly boosters in a prevalence-reduction model, where the prevalence of the disease reduces proportionally to the immunity gap each year and a penalty is imposed for effective immunity being less than the HIT. BoHV-1 vaccination inhibits reactivation of latency and prevents shedding of the virus very effectively [9]; hence, under vaccination, R_0_ was assumed at a lower value of 2, resulting in a HIT value of 50%. When effective immunity equaled or exceeded HIT, the prevalence was slashed by 50% per year. When effective immunity was less than HIT, only a 10% reduction in prevalence per year was made. The results are shown in Figure 2.

From the results of the simulation, it is apparent that periodic re-vaccinations are essential for effective control. At the lowest effective immunity assumed with a combination of 60% coverage and 70% efficacy, prevalence does not reduce to <5% even after 10 years of annual re-vaccination under any of the four initial prevalence scenarios. However, with booster vaccination every six months, prevalence decreases to <1% within five to seven years and <0.1% within eight to ten years in all prevalence scenarios. Predictably, it is also obvious from the results of the simulation that higher levels of vaccine coverage afford faster reductions in the prevalence of the disease. However, it is well known that higher vaccination coverage increases costs of vaccination with diminishing returns on investment [25]. This trade-off between vaccination coverage and vaccination costs is an important policy consideration, particularly for insidious livestock diseases like IBR, and finding a “satisficing” [23] level of vaccine coverage that offers optimum reduction in disease prevalence vis-à-vis return on investment is critical to the success of such programs.

**Figure 3:**
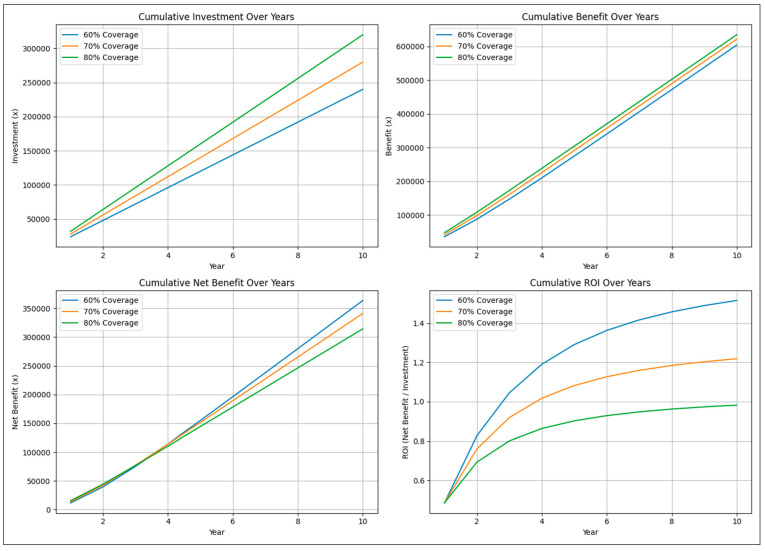
Cumulative investment in vaccination, cumulative benefits from vaccination, cumulative net benefits from vaccination, and cumulative return on investment (ROI) were simulated over a period of 10 years, assuming losses due to the disease at 20*x*, cost of vaccination at 2*x*, and cost of booster/re-vaccination at 1*x*; where *x* is an arbitrary unit of currency. Arbitrary units, instead of absolute values, were used for simplicity of depiction and ease in interpreting the results.

The final simulation modeled the cost–benefit of vaccination against BoHV-1 at three different levels of vaccine coverage, i.e., 60, 70, and 80% in a population of 10,000 animals with an initial IBR prevalence of 33% [10]. Initial vaccine efficacy was assumed at 80% [19] with 5% additive efficacy per booster, capped at 90%. Primary vaccination against IBR requires administration of two doses of the vaccine one month apart, followed by booster revaccinations at every six-month interval. The cost of vaccinating once was assumed at 1*x*, where *x* is an arbitrary unit of currency. Hence, the cost of boosters/re-vaccinations was 1*x*, and that of primary vaccination was 2*x*. Instead of an absolute value, our study assumed losses due to IBR at 20*x*, i.e., 10× the cost of primary vaccination. Arbitrary units, instead of absolute values, have been used for ease of interpreting the results. The results of the simulation are shown in Figure 3.

As expected, cumulative investment accelerates with increasing vaccination coverage. However, the benefits from vaccination accumulate at a constant rate, resulting in an inflexion of the cumulative net benefits obtained with lesser vaccination coverage over the cumulative net benefits obtained with higher vaccination coverage within a few years. This results in marked differences in cumulative ROI. From the results of our simulation, a net positive ROI (i.e., >1) is achieved with 60% and 70% vaccination coverage in less than 4 years, whereas 80% vaccine coverage fails to deliver a net positive ROI even after 10 years. Therefore, aiming for 70% vaccination coverage, which should afford faster reduction in disease prevalence than 60% vaccination coverage, whilst yielding net positive and better ROI than 80% vaccination coverage, may be a more suitable strategy for the control of IBR in India. Although the calculations have been shown for a population size of 10,000 animals and an initial disease prevalence of 33%, the inferences are independent of these assumptions. Further, while our simulations suggest that 70 percent vaccination coverage offers an optimal balance between prevalence reduction and ROI under the modeled conditions, variations in herd size, regional prevalence, vaccine handling, and farm practices could alter the outcomes substantially. For example, the inferences do change if the relative costs of losses due to the disease and those of vaccination change, with a greater difference between the two resulting in a net positive ROI even at 80% vaccine coverage. Similarly, an increase in vaccine efficacy increases the ROI at any given level of vaccination coverage and vice versa. Although milk production losses would be slightly lower due to poorer productivity of Indian cattle, the per cow losses from IBR in India are likely to be similar to those from Turkey, estimated at an average of US$379, i.e., ₹32,000 approx. [5], when corrections for the other factors, including inflation, are made. It follows that 70% vaccination coverage should result in a rapid reduction in IBR prevalence in the country at a favorable ROI, even when the overall cost of vaccinating animals against IBR on each occasion is up to about ₹1600.

The findings of our study are limited by its assumptive nature, as is true for most modeling studies of this kind. Nevertheless, most of the assumptions in this study have been drawn from existing studies under comparable settings. Further, a simple linear deterministic model has been used, which presents a very “average” picture of the problem and several effects such as death and culling of aged and sick animals, introduction of and replacement with new animals, transfer of maternal immunity, re-activation of latency, co-existence of other hosts and reservoir species, DIVA capabilities, intensive vaccination of residual animals, emergence of escape mutants under selection pressure due to vaccinal immunity, sudden or large-scale changes in extant livestock-rearing and herd health management practices, etc. have not been accounted for. A more complex model with stochastic attributes could have been used, although it would have had its own set of limitations, especially towards the application of the findings to a very large and diverse population, where deterministic models tend to perform better than stochastic models [26].

To conclude, our study modeled the protection and cost benefits from vaccinating cattle against Infectious Bovine Rhinotracheitis in India. Given the endemicity of the disease in the country, implementation of proper vaccination practices and appropriate farm biosecurity measures remains essential to achieve herd immunity in the face of realistic levels of vaccination coverage. Re-vaccination at a six-month interval, which imparts strong booster effects to vaccine efficacy and results in significantly less time for reduction in the prevalence of the disease, must be practiced. Lastly, 70% vaccination coverage offers a favorable combination in terms of the rate of reduction in prevalence and ROI. Based on the findings of this study, a vaccination program aiming for primary vaccination with two doses followed by continuing bi-annual re-vaccination with a single dose to achieve 70% vaccination coverage in the cattle population can be recommended for the control of IBR in India.

## Data Availability

The original contributions presented in this study are included in the article/supplementary material. Further inquiries can be directed to the corresponding author.

## Supplementary Materials

The following supporting information can be downloaded at: https://www.mdpi.com/article/10.3390/pathogens14090955/s1, Supplementary File S1: Plotly codes used for calculations and generating graphs.
