# Supplementary material for: A Simple Deterministic Model of Protection and Cost Benefits from Vaccinating Indian Cattle Against Infectious Bovine Rhinotracheitis
Source: Pathogens. 2025 Sep 22;14(9):955. doi: 10.3390/pathogens14090955 (PMC12473101; doi:10.3390/pathogens14090955)
Supplement: Supplementary file 1 [file pathogens-14-00955-s001.zip › pathogens-3851449-supplementary.pdf]

Supplementary File S1

Plotly codes used for calculations and generating graphs

Vaccine coverage

```
import pandas as pd
import numpy as np
import seaborn as sns
import matplotlib.pyplot as plt
# Define R0 values and vaccine efficacy levels
R0_values = [3, 4, 5, 6, 7]
efficacy_levels = [0.7, 0.8, 0.9]
# Create a DataFrame to store vaccine coverage values
coverage_data = pd.DataFrame(index=R0_values, columns=[f"{int(e*100)}%" for e in efficacy_levels])
# Calculate required vaccine coverage using the formula
for R0 in R0_values:
    for E in efficacy_levels:
        Vc = (1 - 1/R0) * (1/E)
        coverage_data.loc[R0, f"{int(E*100)}%"] = round(Vc, 4)
# Display the table
print("Required Vaccine Coverage Table:")
print(coverage_data)
# Generate heatmap
plt.figure(figsize=(8, 6))
sns.heatmap(coverage_data.astype(float), annot=True, cmap="YlOrRd", cbar_kws={'label': 'Vaccine Coverage'})
plt.title("Heatmap of Required Vaccine Coverage")
plt.xlabel("Vaccine Efficacy")
plt.ylabel("$R_0$ Value")
plt.tight_layout()
plt.savefig("vaccine_coverage_heatmap.png")
plt.show()
```

Booster effects

```
import numpy as np
import pandas as pd
import matplotlib.pyplot as plt
import seaborn as sns
# Define parameters
initial_prevalences = [18, 35, 45, 60]
efficacies = [70, 80, 90]
coverages = [60, 70, 80]
years = list(range(11))
HIT = 50  # Herd Immunity Threshold
# Function to calculate prevalence over time
def calculate_prevalence(initial, efficacy, coverage, booster=False):
    prevalence = [initial]
    effective_immunity = efficacy * coverage / 10000
    for year in range(1, 11):
        if booster:
            efficacy = min(efficacy + 10, 95)  # 2 boosters per year, 5% each
        effective_immunity = efficacy * coverage / 10000
        reduction_rate = 0.5 if effective_immunity >= HIT / 100 else 0.1
        new_prevalence = prevalence[-1] * (1 - reduction_rate)
        prevalence.append(new_prevalence)
    return prevalence
# Create data for heatmaps
heatmap_data = []
for booster in [False, True]:
    for initial in initial_prevalences:
        panel_data = []
        for efficacy in efficacies:
            for coverage in coverages:
                prevalence = calculate_prevalence(initial, efficacy, coverage, booster)
                panel_data.append(prevalence)
        heatmap_data.append(np.array(panel_data))
# Plotting
fig, axes = plt.subplots(2, 4, figsize=(24, 10), sharex=True, sharey=True)
fig.suptitle("Year-
wise Disease Prevalence Over 10 Years\n(Top: No Booster, Bottom: With Booster)", fontsize=16)
for i, ax in enumerate(axes.flat):
    sns.heatmap(heatmap_data[i], ax=ax, cmap="YlOrRd", cbar=i % 4 == 3,
                xticklabels=years, yticklabels=[f"{e}-
{c}" for e in efficacies for c in coverages],
                annot=True, fmt=".1f")
    booster_status = "No Booster" if i < 4 else "With Booster"
    initial = initial_prevalences[i % 4]
    ax.set_title(f"{booster_status} - Initial Prevalence: {initial}%")
    ax.set_xlabel("Year")
    ax.set_ylabel("Efficacy-Coverage")
plt.tight_layout(rect=[0, 0, 1, 0.95])
plt.savefig("combined_8_panel_heatmap.png")
plt.show()
```

Cost-benefit Analysis

```
import matplotlib.pyplot as plt
import numpy as np
# Parameters
herd_size = 10000
initial_prevalence = 0.33
vaccine_efficacy_start = 0.80
coverage_levels = [0.60, 0.70, 0.80]
vaccine_cost = 2
booster_cost = 1
loss_per_case = 20
years = 10
# Initialize storage
cumulative_investments = {c: [] for c in coverage_levels}
cumulative_benefits = {c: [] for c in coverage_levels}
cumulative_net_benefits = {c: [] for c in coverage_levels}
cumulative_rois = {c: [] for c in coverage_levels}
# Simulation
for coverage in coverage_levels:
    prevalence = initial_prevalence
    cumulative_investment = 0
    cumulative_benefit = 0
    cumulative_net_benefit = 0
    efficacy = vaccine_efficacy_start
    investment_per_year = herd_size * coverage * (vaccine_cost + 2 * booster_cost)
    for year in range(1, years + 1):
        # Update efficacy with booster (5% every 6 months, capped at 90%)
        efficacy = min(efficacy + 0.10, 0.90)
        # Calculate new prevalence
        reduction_factor = efficacy * coverage
        prevalence *= (1 - reduction_factor)
        infected_animals = prevalence * herd_size
        # Calculate benefit and investment
        benefit = (initial_prevalence * herd_size - infected_animals) * loss_per_case
        cumulative_benefit += benefit
        cumulative_investment += investment_per_year
        cumulative_net_benefit = cumulative_benefit - cumulative_investment
        roi = cumulative_net_benefit / cumulative_investment if cumulative_investment > 0 else
 0
        # Store values
        cumulative_investments[coverage].append(cumulative_investment)
        cumulative_benefits[coverage].append(cumulative_benefit)
        cumulative_net_benefits[coverage].append(cumulative_net_benefit)
        cumulative_rois[coverage].append(roi)
# Plotting
fig, axs = plt.subplots(2, 2, figsize=(14, 10))
years_range = list(range(1, years + 1))
# Panel 1: Cumulative Investment
for coverage in coverage_levels:
    axs[0, 0].plot(years_range, cumulative_investments[coverage], label=f'{int(coverage*100)}%
 Coverage')
axs[0, 0].set_title('Cumulative Investment Over Years')
axs[0, 0].set_xlabel('Year')
axs[0, 0].set_ylabel('Investment (x)')
axs[0, 0].legend()
axs[0, 0].grid(True)
# Panel 2: Cumulative Benefit
for coverage in coverage_levels:
    axs[0, 1].plot(years_range, cumulative_benefits[coverage], label=f'{int(coverage*100)}% Co
verage')
axs[0, 1].set_title('Cumulative Benefit Over Years')
```

```
axs[0, 1].set_xlabel('Year')
axs[0, 1].set_ylabel('Benefit (x)')
axs[0, 1].legend()
axs[0, 1].grid(True)
# Panel 3: Cumulative Net Benefit
for coverage in coverage_levels:
    axs[1, 0].plot(years_range, cumulative_net_benefits[coverage], label=f'{int(coverage*100)}
% Coverage')
axs[1, 0].set_title('Cumulative Net Benefit Over Years')
axs[1, 0].set_xlabel('Year')
axs[1, 0].set_ylabel('Net Benefit (x)')
axs[1, 0].legend()
axs[1, 0].grid(True)
# Panel 4: Cumulative ROI
for coverage in coverage_levels:
    axs[1, 1].plot(years_range, cumulative_rois[coverage], label=f'{int(coverage*100)}% Covera
ge')
axs[1, 1].set_title('Cumulative ROI Over Years')
axs[1, 1].set_xlabel('Year')
axs[1, 1].set_ylabel('ROI (Net Benefit / Investment)')
axs[1, 1].legend()
axs[1, 1].grid(True)
plt.tight_layout()
plt.savefig("cumulative_economic_metrics.png")
plt.show()
```
